# Motivations for nutrition information-seeking behavior among Belgian adults: a qualitative study

**DOI:** 10.1186/s12889-022-14851-w

**Published:** 2022-12-27

**Authors:** Jules Vrinten, Kathleen Van Royen, Sara Pabian, Charlotte De Backer, Christophe Matthys

**Affiliations:** 1 Department of Chronic Diseases and Metabolism, Clinical and Experimental Endocrinology, KU Leuven, Herestraat 49, box 7003, 3000 Leuven, Belgium; 2 Faculty of Social Sciences, Dept. of Communication Sciences, University of Antwerp, Stadscampus – Building M, St-Jacobstraat 2, 2000 Antwerp, Belgium; 3 Research Centre The Cycle of Care, Karel de Grote University College, Brusselstraat 45, 2018 Antwerp, Belgium; 4 Tilburg University, Tilburg, School of Humanities and Digital Sciences, Tilburg Centre for Cognition and Communication, PO Box 90153, 5000 LE Tilburg, The Netherlands; 5 Department of Endocrinology, University Hospitals Leuven, DIEET – Herestraat 49, Leuven, 3000 Belgium

**Keywords:** Nutrition, Information-seeking behavior, Qualitative, Motivation, Media

## Abstract

**Background:**

Nutrition information-seeking behavior is highly prevalent even though it can be challenging to find reliable nutrition information in the current media landscape. Previous quantitative research has identified which population segments use which sources, yet little is known about motivations underpinning nutrition information-seeking behavior. Understanding motivations for seeking nutrition information can increase the efficacy of future nutrition education efforts. The present study aims to identify motivations for nutrition information-seeking behavior among Belgian adults.

**Methods:**

In-depth qualitative interviews were conducted with 20 adults (*n* women = 15, *n* men = 4, *n* other = 1). Audio-recorded interviews were transcribed verbatim and analyzed in NVivo 12 using inductive thematic analysis. The coding process involved open and axial coding combined with constant comparison to identify themes.

**Results:**

The interviews revealed a diverse spectrum of motivations for nutrition information-seeking behavior. Five categories of motivations centered on health management, cognitive needs, affective needs, social integrative needs, and personal identity. Participants indicated seeking nutrition information to regain a sense of control over their health and it also helped them express their autonomy. Additionally, participants sought nutrition information out of curiosity or out of a long-standing interest. Nutrition information-seeking was also used as a form of emotion regulation with participants actively engaging in seeking behavior to pursue enjoyment, diversion, confirmation, inspiration, and even relaxation. Furthermore, nutrition information-seeking enabled participants to meet social integrative needs by helping them connect to others, gather social support, help others, and sometimes convince others. Lastly, participants indicated a need for nutrition information to express or defend their identity.

**Conclusions:**

Our results indicate that beneath the surface of a more apparent need for nutrition information lies a range of motivations demonstrating that nutrition information serves more complex needs than simply information needs. To improve the efficacy of future nutrition education efforts, communication strategies must be tailored to a diverse range of motivations.

**Supplementary Information:**

The online version contains supplementary material available at 10.1186/s12889-022-14851-w.

## Background

Health communicators increasingly struggle with disseminating evidence-based nutrition information in a media landscape filled with unreliable and contradictory nutrition information [1, 2]. One way to maximize health communication efforts could be by tailoring communication strategies to individuals’ needs [3]. Needs are key factors that determine whether and how people gather, process, and apply information [4]. Needs influence and shape the entire information-seeking process and contribute to differential media effects [5, 6]. Therefore, a comprehensive understanding of needs driving information-seeking behavior is required to develop effective communication strategies.

Current research in the field of nutrition information-seeking behavior (NISB), which can be defined as actively seeking nutrition information via on- and offline media, has discovered that the Internet is a popular source of nutrition information and that especially women and higher-educated people seek nutrition information [7, 8]. However, little is known on needs driving nutrition information behavior.

To investigate needs driving information behavior, several research traditions are of relevance. First, from an information science perspective (for a comprehensive overview see Case [9]), it has been argued that the term “information needs” is misleading and that a need for information sprouts from a need to fulfill more basic human needs [4]. Based on works in the field of psychology, three categories of human needs are distinguished being physiological (e.g. nutrition, shelter), affective (e.g. need for attainment), and cognitive (e.g. need to plan, learn a skill). Equally, the salience of a gap has been recognized as a starting point for information seeking [10]. This gap hypothesis has been expanded upon by Dervin who takes a sense-making approach by arguing that an information need derives from a user’s desire to make sense of a current situation using whatever the user considers to be information [11]. Under this sense-making approach, users described several uses for information such as companionship, support, pleasure, and a new way of looking at things [12]. Closely related to Dervin’s sense-making approach is the work of Kulthau. According to Kulthau, uncertainty and anxiety reduction form the primary motivators of information seeking [13]. Kulthau further emphasizes the importance of affective states as users progress through their search process and advocates a holistic view on information seeking. Second, a conceptually related field of research is the field of health information-seeking behavior (HISB). Studies within the domain of HISB suggest that people seek online health information to reduce uncertainty, out of a need for acknowledgement and perspective, to supplement their knowledge, for the added benefit of online anonymity, and to bypass barriers associated with traditional sources of health information [14–18]. Third, from a mass communication perspective, uses and gratifications theory (UGT) has an extensive history of examining needs that drive media use [6].

Within UGT, the audience is viewed as actively selecting and using media to fulfil psychological and social needs [6], which corresponds to the active nature of NISB. Uses and gratifications theory has previously been used in the context of health information-seeking behavior via websites [19], magazines [20], and YouTube [21], but has not yet been applied to the specific context of nutrition.

The aim of this study was to explore motivations for NISB from a media use perspective, specifically UGT.

## Materials and methods

A qualitative study design has been used and the consolidated criteria for reporting qualitative research (COREQ) [22] were used in the reporting of this study (Additional file 1).

### Participants and recruitment

A convenience sample was recruited using Facebook between January 24th and March 9th, 2020. Recruitment messages were posted in public groups both nutrition-related and of general interest. Participants were offered a 10-euro incentive for participation. Eligibility was evaluated by use of a brief online survey in which age, gender, and frequency of information-seeking behavior were measured. Individuals were considered eligible if they were 18 + years of age and sought nutrition information once a month or more often. Eligible participants were contacted via email for an interview.

### Interview guide

A semi-structured interview guide was developed and pre-tested for clarity with two individuals prior to the study. The guide contained several questions regarding the nutrition information-seeking process with three questions specifically pertaining to motivations for NISB. In the opening part of the interview participants were asked: 1) why they were interested in nutrition; 2) why they sought information on this topic; and 3) if they sought this information for themselves or for others. The last question was added since in the context of health information, people often seek information for others [23]. The second and third questions were repeated for several nutrition-related topics (e.g., carbohydrates, gluten, sustainable nutrition, vegetarianism) to examine whether motivations differed per topic. Given the interconnectedness of human and planetary health [24], in this study, nutrition information was defined as all information pertaining to a healthy and/or sustainable diet. Furthermore, it was stressed that this did not include recipes. The interview guide and topics list are available from the corresponding author on request.

### Procedure

The semi-structured, in-depth interviews were conducted between March and April 2020. All interviews were conducted by the first author who has experience in qualitative data collection. Two weeks before the interview, participants received additional information about the study and were asked to keep a diary of their NISB indicating 1) topics, 2) media channels and 3) motivations. It was emphasized that information-seeking behavior was limited to *active* behavior (to exclude information *scanning* i.e. passive exposure to information during routine media use [25]) and to seeking *information* (to exclude, e.g., recipes). Recipes were excluded because these do not primarily focus on nutrition, which can be defined as “the science of the nutrients in foods and their actions within the body” [26] (p. 3). It was also stressed that all media sources (both online and offline) were relevant. This diary was used as a mnemonic during the interview and was not considered part of the dataset. Interviews were conducted face-to-face (*n* = 6), via video call (*n* = 12) or via audio-only call (*n* = 2). Interviews were audio-recorded and transcribed verbatim. All identifying information was removed from the transcripts to ensure anonymity. Field notes were made directly after each interview and data was collected until saturation was reached. Data saturation was assessed during data collection based on researcher field notes.

### Data analysis

Interviews were transcribed verbatim and anonymized. Qualitative data analysis was performed in NVivo 12 using an inductive thematic analysis method [27]. Investigator triangulation (i.e. the use of multiple observers to account for subjective influences on data interpretation) was used to increase validity of results [28]. The analysis process consisted of six phases [29]. In the first phase, the first author regained familiarity with the data by thoroughly reading and re-reading all transcripts. In the second phase, initial codes were generated by the first and second authors who independently coded the first interview using an open coding approach followed by axial coding. In the third phase, the same two researchers independently collated codes into themes and subthemes thus creating two independent coding schemes. These coding schemes were extensively discussed and adapted until a preliminary coding scheme was agreed upon. The construction of this coding scheme was mainly data-driven but motivations for media use derived from UGT [30] were used as sensitizing concepts. Additionally, two concepts from the field of educational psychology were added to the coding scheme: interest and curiosity. Interest is defined as “an ongoing and deepening relation of a person to particular subject content” [31]. Curiosity is defined as a psychological state that is activated when a person becomes aware of a knowledge gap and believes this gap can be reduced [32]. To conclude the third phase, all remaining interviews were coded by the first author using the initial coding scheme. In the fourth phase, the initial coding scheme was reviewed and refined. Constant comparison was used to check the data for emerging themes and to verify a good fit with the coding scheme. The coding scheme was adapted throughout the coding process until a final coding scheme was constructed in the fifth phase. To ensure clarity of the themes, the final coding scheme was discussed with the fourth author. In the sixth and final phase, descriptions of themes and subthemes were provided along with quotes to illustrate their conceptualizations. Quotes were originally in Dutch but were translated backwards with the help of a native English speaker.

## Results

A total of 42 people met the eligibility criteria and were invited for participation. Of the 42 eligible candidates, twenty took part in the study. The sample consisted primarily of highly educated women (*n* = 15) aged 26–50 years who sought nutrition information once a week or more (Table 1). The interviews ranged in duration between 51 and 66 min with an average of 58 min. Categories of motivations for NISB emerging from the data were health management, cognitive needs, affective needs, social integrative needs, and personal identity. Even though there was considerable overlap between the various categories, each motivation was discussed in the subsection deemed most appropriate. Motivations were aggregated across different nutrition topics since the data showed insignificant variation over topics. To identify participants’ contributions, each quote includes an identification number.

**Table 1:** Characteristics of participants (n 20)

		***N***
*Gender*	Women	15
	Men	4
	Other	1
*Age*	18—25 years	1
	26—50 years	15
	51 – 75 years	4
*Education*	Primary	0
	Secondary	5
	Bachelor’s degree	5
	Master’s degree	9
	Doctorate	1
*Frequency of nutrition information seeking*	More than once week	9
	Once a week	6
	Once a month	5

### Health management

In terms of health management, three motivators for NISB were identified: health improvement, autonomy, and prevention of health issues. Health improvement emerged as an often-mentioned motivator for NISB. This may be unsurprising given that little over half of participants reported a medical history (*n* = 12). Health improvement as a motivator for NISB was intricately linked to a need for autonomy. By seeking nutrition information, participants regained a sense of control over their health as exemplified by participant 11 “I really felt like I could heal myself without constantly having to take more and more pills.” Another participant with a history of serious health issues remarked:

One of the things that I did then, apart from all the treatments and so on, was to go into that [nutrition] because I was just convinced that that was one of the things that I could do myself to try and get my health back in order. . . To be able to do something as well. Apart from the doctors who did things for me, I wanted to do something myself. (Participant 12)

The need for autonomy was accompanied by an internal locus of control, with participants believing “that one stays or becomes healthy or sick as a result of his or her behavior” [33]. Participant 13: “My interest in nutrition comes mainly from my own value of living healthy... and keeping my body healthy.” Participants also sought nutrition information to aid in the prevention of health issues. Most participants (*n* = 14) had encountered health problems because of the diagnosis of a health problem in themselves or in relatives. This had led to a heightened sense of susceptibility, which resulted in an urgency to prevent potential future health issues. Participant 9 stated “I'm 26 now, but I'm also trying to avoid illnesses or later illnesses. My mum's friend has type 2 diabetes and those are things I wouldn't worry about yet, but I actively want to avoid them at this stage of my life.” Another participant alluded to the preventative power of nutrition “Because my family also has a fairly high incidence of the hereditary type of diabetes, and one of the benefits of intermittent fasting is that your insulin level actually remains stable for a long time.” (Participant 19).

### Cognitive needs

Since gathering information is primarily a cognitive matter, cognitive needs naturally emerged as key motivations for seeking nutrition information. To obtain a more detailed understanding of cognitive needs, a distinction was made between curiosity and interest. The latter was one of the most frequently mentioned motivations for NISB. Participants indicated having a long-standing interest in nutrition, the origin of which was not always clear “I’ve actually always had an interest in nutrition” (Participant 14). Another participant even called NISB her “hobby” (Participant 2). Participants expressed great interest in continuously learning about nutrition and over the years, this interest had become an integral part of their identity. Curiosity was another frequently mentioned motivator for NISB. Based on the interview data, it was clear that curiosity was aroused when knowledge gaps became salient. Curiosity was especially aroused during information scanning, subsequently triggering active information-seeking behavior. Participant 5 illustrated “I mostly seek information about nutrition precisely because I come across it somewhere and then I want to know what it is.” Furthermore, during active nutrition information seeking, questions would arise triggering more NISB, with one participant stating, “When I read something, other questions arise in my head and then I have to investigate those other questions as well.” (Participant 2). Information seeking was perceived as a continuous process that was never finished. It seemed that to trigger information-seeking behavior, more was needed than just the saliency of an information gap and the belief that this gap could be reduced. The topic needed to be personally relevant, interesting and participants needed to be convinced that obtaining this information would benefit them and/or their surroundings. If these conditions were met, participants reported spending more time on information seeking.

### Affective needs

Even though participants often did not explicitly mention emotion regulation as an initial motivation for NISB, it was clear that information seekers deliberately used NISB to fulfil certain affective needs. Five affective needs were distinguished: a need for enjoyment, relaxation, inspiration, diversion, and confirmation. By far the most frequently experienced emotion was a sense of enjoyment. Participants enjoyed gathering knowledge and having their curiosity satisfied. Participant 12 said, “It's always nice to know more. So that makes me feel good and I like to learn things anyway.” The fulfilment of other needs (e.g., cognitive needs, need for autonomy and social integrative needs) was a prerequisite for the elicitation of these positive affective states. Another need driving nutrition information seeking was the need for inspiration. For example, one participant indicated feeling inspired by reading blogs of people with similar values and beliefs (participant 13). Another participant made a distinction between applicable and inspirational information, with participant 6 remarking, “I was actually looking for information, but more as inspiration.” More in the domain of information scanning, relaxation and diversion were frequently mentioned motivators for NISB. Participants indicated that scrolling through social media feeds brought a sense of relaxation and that they used information seeking/scanning as a time filler and escape from everyday life. Participant 10 indicated, “As soon as I get bored, I look at a few stories and there is a lot about nutrition and sport.” Another affective need was a need for confirmation. Participants often suffered from doubts about the reliability of information found and credibility of the source. Participant 13 illustrated this by remarking “Just having to constantly ask myself, is it true, what is it based on?” These doubts led to a continued information search to reduce uncertainty “I always have the feeling that I have to keep looking for information. There is hardly ever one answer, a precise answer.” (Participant 2) Confirmation was also used as an ego boost. Participants sought information that confirmed their behaviors, cognitions, values, attitudes, and beliefs. Participant 11 said, “That I can go to this platform and read... it is all correct what I have read.” Positive affective states were elicited when confirmation was obtained.

### Social integrative needs

Nutrition information seeking seemed to be driven by four social integrative needs: a need for connection, social support, helping others, and convincing others. First, our participants were part of social circles in which nutrition information was regularly exchanged. Seeking and sharing nutrition information thus served to strengthen social connections which in turn fulfilled an underlying need for connection*.* Additionally, the exchange of nutrition information functioned as a trigger for further information seeking, with participant 7 indicating, “Sometimes my mum sends me an article or a video she’s seen from someone who has posted something and then I take it from there.” Second, seeking and sharing nutrition information was driven by a need for social support. Participants indicated a need for social support to sustain or alter their nutrition-related behavior, with participant 8 saying, “There are some who are very focused on this in my circle of friends and exchange a lot of information... to actually encourage each other a little bit and share the information we have.” Third, participants often engaged in NISB to help others. Participants often functioned as key information providers within their social networks and enjoyed helping others with participant 9 remarking, “Most of my friends and family know I'm involved in it... they also know they can come to me to ask questions and I will be happy to look it up with them.” Finally, some participants opted for an activist approach and needed nutrition information to convince others to alter their dietary behaviors. Within the context of sustainability, participant 16 illustrated this by stating: “Because I’m specifically looking for arguments to ban the purchase of Oreo biscuits and to convince not only my family members but also my friends to stop buying and eating those and I actually do that!”.

### Personal identity

Regarding personal identity, two motivators for NISB were distinguished: identity expression and identity defense. Nutrition information seeking aided in identity expression by reinforcing values and beliefs. The reinforcement of values and beliefs led to a strengthened sense of self, which empowered participants to behave more in line with their values. For example, participant 4 deliberately sought information to reaffirm his beliefs on alcohol consumption to aid him in alcohol abstinence, “Tourné mineral [local awareness campaign on alcohol use] started again and I thought I'll stop completely again. And that's why I started looking for more information about it. I'm very concerned about what it does to your body. And that's a lot actually.” To translate values into behavior, participants also used a variety of aids, such as diet apps, apps with information on E-numbers, and calendars of seasonal vegetables. Another motivation regarding personal identity was identity defense. Participants sought arguments to win future discussions and needed convincing information to persuade others “If my parents start talking about it [her consumption of diet soft drinks] that I can actually say something back.” (Participant 10) Another participant needed information to defend her choice not to eat eggs “I had when I stopped eating eggs a long time ago, that people asked "well, why do you stop eating eggs, because a chicken lays an egg every day anyway, doesn't it? And there's nothing wrong with that?”” (Participant 14).

**Fig. 1:**
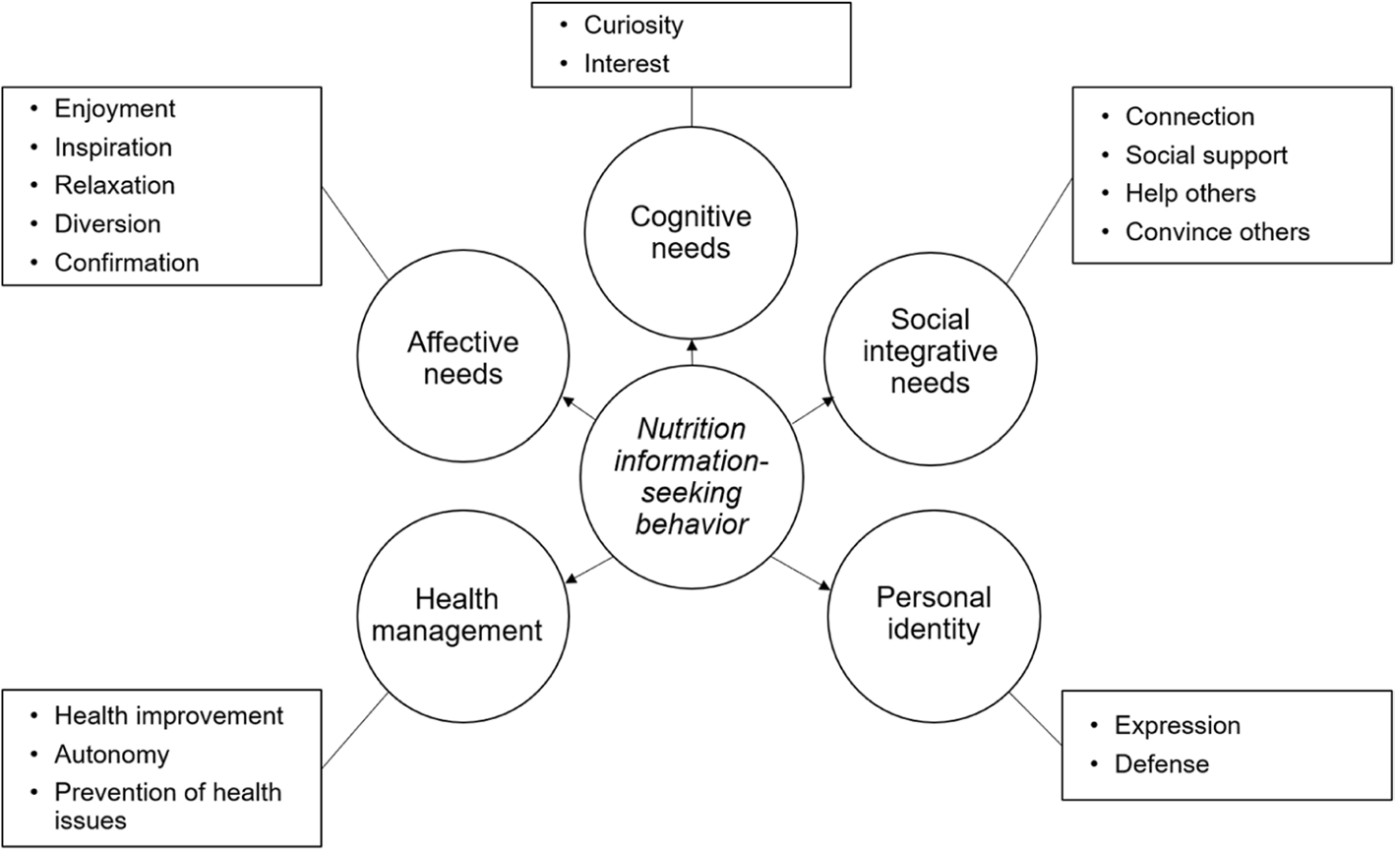
Overview of motivations for nutrition information-seeking behavior

To summarize, five categories of motivations for NISB were distinguished: health management, cognitive needs, affective needs, social integrative needs, and personal identity (Fig. 1). Our findings thus suggested there was a diverse range of motivations driving NISB.

## Discussion

This study is novel in its approach by extending applications of UGT from the context of health information seeking to the nascent research field of NISB. Previous UGT research recognized information gathering, identity formation, social contact, and entertainment as motivations for media use [34]. The current study expands UGT by including concepts from (educational) psychology (i.e., interest, curiosity, locus of control, autonomy) and by distinguishing novel motivations such as health management and a need for confirmation. By employing a qualitative methodology, this study provides a deeper understanding of what motivates individuals to seek nutrition information, which is crucial for health communication professionals seeking to develop tailored communication strategies.

Five categories of motivations for NISB were distinguished: health management, cognitive needs, affective needs, social integrative needs, and personal identity. Health management emerged as an often-mentioned motivator for NISB. Participants expressed a need for nutrition information to improve their health and to prevent future health issues. The fact that people turn to nutrition can be explained by well-researched associations between dietary behavior and health outcomes [35]. Little over half of participants had a medical history which fostered nutrition information seeking. The presence of health problems might have increased their perceived health risk, which has been associated with increased health-related information seeking [36]. Arguably, an increased perceived health risk could have been concomitant with anxiety leading participants to seek information as a means of anxiety reduction. This would be in line with the work of Kulthau who recognized anxiety reduction as key motivator for information seeking [13]. Additionally, participants displayed an internal locus of control, which has been linked to higher levels of health information seeking [37]. Another motivator in the context of health management was a need for autonomy with participants expressing a need to feel in control of their health. Autonomy is recognized within self-determination theory as one of three basic human needs, along with competence and relatedness [38]. Prior research has found that supporting autonomy contributes positively to physical and psychological health [39]. This need for autonomy resembles prior research stating the role of information seeking as a means for self-actualization and self-control [11]. Future communication strategies could aid in the fulfilment of this need by offering nutrition information in an autonomy-supportive way. For example, by using non-controlling language, offering choices, and emphasizing personal responsibility [40]. Other behavior change techniques that support autonomy are providing a meaningful rationale (e.g., by informing people about health consequences of dietary behaviors), setting graded tasks and goal setting [41].

Regarding cognitive needs, interest and curiosity have been withheld as separate motivations for NISB, which is an extension to UGT. The finding that interest emerged as a motivator for NISB may be related to our recruitment method in which we specifically sought individuals with an interest in nutrition. Interest can also be viewed as a form of enduring involvement, which in the context of health has been coined “health consciousness” [42]. People with a high level of health consciousness are intrinsically motivated to stay or become healthy and to actively seek health information [42, 43].

The finding that curiosity (i.e. the experience of a gap in knowledge combined with the belief that this gap can be reduced) incited information seeking echoes notions from the field of information science where the experience of a gap has been distinguished as precursor of information seeking [10, 12]. In the context of health information seeking, curiosity has equally been recognized as a motivator of information seeking, with curiosity often being sparked during passive information scanning [44]. Our findings indicated that certain conditions needed to be met for participants to transition from a passive information *scanning* process to an active information *seeking* process. Specifically, participants needed to believe that obtaining this information would benefit them and/or their surroundings and the information needed to carry personal significance. These findings are in line with the comprehensive model of information seeking which distinguishes positive efficacy beliefs and personal relevance as antecedents of information seeking [20]. Another model that is relevant in this context is the elaboration likelihood model (ELM) [45]. The ELM proposes two routes of information processing: a central route and a peripheral route. Central route processing occurs when people are motivated and capable. This type of information processing results in stronger and more durable attitudes and behavior changes. Alternatively, information can be processed via the peripheral route, which is associated with minimal cognitive effort, is more reliant on heuristics and has only a short-standing impact on attitudes and behavior changes. It has been suggested that information scanning leads to peripheral route processing and information seeking to central route processing [46]. Therefore, to maximize the effect of nutrition communication on dietary behavior, future communication strategies should focus on triggering information seeking (central route processing) during routine information scanning (peripheral route processing). Encouraging central route processing can be achieved by enhancing either individuals’ motivation or capabilities for information processing. Motivation can be enhanced by tailoring messages such that they become personally relevant to the recipient [47]. For example, the Netherlands Nutrition Centre has a popular mobile app in which people can track their dietary behavior and personalized advice is offered based on this information [48]. Additionally, messages can be shaped in a way that maximize the ability of the recipient, such as being easy to understand offered without distractions, and self-paced [47]. Regarding the first point, there is evidence to suggest that the reading level of nutrition information materials exceed recommended reading levels for public health communication [49, 50]. In Flanders, Belgium where this study was conducted, 15% of adults are low literate, meaning they struggle with basic literacy skills necessary for optimum personal development and full participation in society (e.g. reading, writing) [51]. Additionally, low literacy is associated with poor health outcomes [52] further highlighting a need to target public health promotion efforts at low literate adults. Regarding the second point, research has shown that reading comprehension plummets when people are distracted [53]. It has been suggested that especially the architecture of the digital environment hinders deep learning by rapidly exposing people to small bits of information embedded within a distracting context [54].

Another way in which the ability of the recipient can be maximized is by increasing their level of critical nutrition literacy. Critical nutrition literacy refers to skills related to the critical appraisal of nutrition information [55] and it is imperative to effectively contextualize scientific evidence. Not all scientific evidence is created equal [56], meaning it is up to individuals to separate fact from fiction. By enhancing critical appraisal skills, individuals become empowered to do this and to decide for themselves the influence novel nutrition information has on their dietary behavior. Thus, given the importance of autonomy-supportive communication, improving critical nutrition literacy could be a key objective of future communication strategies. However, there is currently a need for research that can focus efforts on improving critical nutrition literacy.

Enjoyment, inspiration, relaxation, diversion, and confirmation were distinguished as affective needs driving nutrition information seeking. These findings, except for a need for inspiration, are in line with earlier UGT research studying motivations of health-related YouTube use [21]. Additionally, these motivators are akin to those recognized in earlier sense-making research [11]. It is surprising to find relaxation and diversion as motivators for NISB given that the focus of this research was on *active* information-seeking behavior. Within UGT, a distinction is made between ritualized and instrumental media use [57]. Ritualized media use is driven by motivations such as diversion, passing time, habit, and relaxation. This contrasts with instrumental media use which is goal-directed, utilitarian, and aimed at satisfying informational needs. In the context of active nutrition information seeking, one would expect to primarily find instrumental media use. One possible explanation for the abundance of ritualized media use in the interviews is the notion that information scanning is far more prevalent than information seeking [58]. Even though this was not the focus of this research, participants reported that information seeking often stemmed from information scanning. It is conceivable that when participants indicated seeking nutrition information to relax or as a diversion, they were in fact *scanning* nutrition information. However, the high prevalence of information scanning is not necessarily negative: scanned information still has a positive impact on individuals’ health behaviors and knowledge [59], which might be explained by peripheral route processing [47]. Future communication strategies could use this knowledge by widely disseminating content optimally suited for information scanning. For example, by developing content that uses experts, which will cue the “expert heuristic” and can result in short-term persuasion [47]. More research is needed to examine the influence of nutrition information scanning on nutrition knowledge and subsequent dietary behavior.

Participants also expressed a need for confirmation, which may reflect the current media landscape in which there is an abundance of conflicting nutrition information [1]. The latter can lead to feelings of confusion and backlash in some individuals [60]. Need for confirmation can also be considered the polar opposite of uncertainty reduction, which has been recognized as a key motivator for information seeking [13].

Nutrition information seeking also fulfilled several social integrative needs. First, participants sought and shared nutrition information to fulfil a need for connection. Additionally, sharing nutrition information also encouraged subsequent nutrition information seeking. Arguably, seeking and sharing information are two different matters, but as our data and previous research in the field of information science show, the concepts are heavily intertwined in the context of information seeking [9]. Furthermore, prior research has shown that social exchange of information makes people more aware of the importance of health-related behaviors, leading them to seek more information [43]. Second, nutrition information seeking, and sharing was driven by a need for social support in sustaining or altering nutrition-related behavior. This finding is consistent with prior research applying UGT to the context of health information seeking [19, 21] and to research in the field of sense-making [11]. Social support has been linked to favorable dietary behaviors through the enhancement of self-efficacy [61] and is therefore recognized as an effective behavior change technique [41]. Future communication strategies could aid in the mobilization of social support by including sharing functionalities that facilitate peer education, strengthen existing social networks and promote the formation of new social networks [62]. For example, several nutrition-related apps currently on the market already have integrated functionalities to strengthen existing and form new social networks [63]. Users of these apps can share their achievements with friends via social media (e.g. Facebook) and can create new connections with other users of the app. Lastly, our participants sought nutrition information to help and/or convince others, which is in line with previous research concerning health information seeking [23].

Regarding personal identity, the main motivations for nutrition information seeking were identity expression and identity defense. Regarding identity expression, participants sought nutrition information to reinforce their values and beliefs. This led to a strengthened self, which in turn facilitated identity expression. These results echo prior research by recognizing the role of information seeking as means to gain self-control and to facilitate self-expression and self-actualization [4, 11]. Online media is especially attractive for seeking reinforcement since complex algorithms tailor information to people’s needs, which may foster false beliefs if individuals are steered toward incorrect information [64]. It is known that novel information should not be too discrepant from the recipient’s beliefs to maximize its persuasive effect [62]. Therefore, for future communication strategies, it may be a balancing act to provide nutrition information that is both evidence-based and not too discrepant from incorrect pre-existing beliefs. A possible way of achieving this is related to the aforementioned method of tailoring: current beliefs can be assessed before novel nutrition information that lies within the realm of acceptability is offered. The second motivation related to personal identity was identity defense, where participants showed an increased need for nutrition information when they felt their identity was under threat. This is not surprising, as some participants alluded to making dietary choices that could be considered deviant from the norm (e.g., abstaining from eggs, alcohol, or meat). Given the cultural significance of dietary choices and the importance of social conventions around food [65], people that deviate from the norm can experience social pressure to defend their choices [66]. Future communication strategies could help people who aspire to healthy and sustainable diets by providing them with coping strategies to deal with possible negative reactions from their social contacts.

### Strengths and limitations

This is a first qualitative study aimed at offering an in-depth understanding of motivations for nutrition information-seeking. Additionally, this study is novel in its application of UGT to nutrition information-seeking behavior. Findings from this study can lay the foundation for the development of tailored communication strategies aimed at disseminating high-quality nutrition information. Limitations of this study include the use of convenience sampling, which led to an overrepresentation of higher educated women. Moreover, by purposely recruiting people that regularly seek nutrition information, the data can be considered biased towards heavy seekers of nutrition information. Perhaps in another sample, other motivations such as the attainment of body ideals or practical needs (e.g. how to eat healthy on a limited budget) could have been recognized as drivers for NISB. Additionally, given the traditional role of women as food providers, the bias towards women may have influenced the importance of social integrative needs as drivers for NISB. Furthermore, a substantial number of participants indicated following non-standard diets, which may have influenced the finding of identity defense as driver for NISB. This means that, as is common in qualitative research, our research findings are not representative for the entire population, but are merely reflections of motivations of nutrition information seeking in a specific subset of the population. Future studies could include a more representative sample to gather a comprehensive view of NISB among different genders (including men) and people of lower education levels. Additionally, motivations for NISB were not asked per type of media even though there is evidence that media type can influence motivations and even create needs [67]. Future studies could remedy this limitation by focusing on one medium, or on several types of media with similar affordances to limit differential influences of media type. Finally, a large part of this research has been conducted during the early days of the COVID-19 pandemic in Belgium. This may have led to a bias in the results with a possible increased importance of health management as a motivation for NISB. Other motivations such as social integrative needs and affective needs may also have played a more significant role, with participants displaying a larger need for connection and for emotion regulation during such stressful times.

## Conclusions

The present study used a qualitative approach to explore underlying needs driving NISB from a UGT perspective. Findings suggest NISB is driven by motivations such as health management, personal identity, and cognitive, affective, and social integrative needs. Future health communication strategies should pay more attention to underlying motivations other than only those related to information gathering to positively influence people’s nutrition knowledge.

## Supplementary Information

**Additional file 1.**

## Data Availability

The interview guide and topics list are available from the corresponding author on request. The datasets used and/or analyzed during the current study are not publicly available. They are available from the corresponding author on reasonable request, subject to approval from the ethics committee that approved the study.
